# Neutrophil granulocyte-dependent proteolysis enhances platelet adhesion to the arterial wall under high-shear flow

**DOI:** 10.1111/j.1538-7836.2010.03890.x

**Published:** 2010-07

**Authors:** N WOHNER, Z KERESZTES, P SÓTONYI, L SZABÓ, E KOMOROWICZ, R MACHOVICH, K KOLEV

**Affiliations:** * Department of Medical Biochemistry, Semmelweis UniversityBudapest, Hungary; † Institute of Nanochemistry and Catalysis, Chemical Research Center, Hungarian Academy of SciencesBudapest, Hungary; ‡ Department of Vascular Surgery, Semmelweis UniversityBudapest, Hungary

**Keywords:** collagen, neutrophil granulocytes, platelets, proteases, proteoglycans, vascular media

## Abstract

*Background:* Under high shear stress platelets adhere preferentially to the adventitia layer of the arterial vessel wall in a von Willebrand factor (VWF)-dependent manner. *Objective:* The present study was undertaken in an attempt to characterize the structural background of the relative thromboresistance of the media and the impact of neutrophil leukocyte-derived proteases (matrix metalloproteinases, neutrophil elastase) on platelet adhesion in this layer of the arteries. *Methods and results:* Platelet adhesion to cross-sections of the human iliac artery was monitored by indirect immunofluorescent detection of GpIIb/IIIa antigen. Exposure of the vessel wall to activated neutrophils or neutrophil-derived proteases increased platelet adhesion to the media about tenfold over the control level at 3350 s^−1^ surface shear rate. In parallel with this enhanced thrombogenicity morphological changes in the media were evidenced by atomic force microscopy (AFM) and scanning electron microscopy (SEM). The fine proteoglycan meshwork seen with Cupromeronic Blue enhancement of the SEM images was removed by the proteolytic treatment and the typical collagen fiber structure was exposed on the AFM images of the media. *Conclusion:* Through their proteases activated neutrophils degrade proteoglycans, unmask VWF binding sites and thus abolish the thromboresistance of the media in human arteries.

## Introduction

Platelets are essential for normal hemostasis and their function is particularly relevant to arrest bleeding in the small arterioles of 10–50 μm in diameter, where the wall shear rate varies from 500 to 5000 s^−1^, representing the highest values in normal circulation [1]. Under these conditions the initial platelet recruitment is von Willebrand factor (VWF)-dependent; platelet adhesion is initiated by the binding of platelet GpIb to VWF immobilized on the thrombogenic surface [2–4]. This interaction slows down platelets sufficiently enough to enable GpVI-collagen-mediated platelet signaling which leads to the activation of GpIIb/IIIa, a receptor required for platelet aggregation [5]. VWF circulates in plasma in a multimeric form containing a variable number of subunits, distinct domains of which bind platelet surface glycoproteins GpIb and GpIIb/IIIa, as well as collagen types I, III and VI [6–8]. The vessel wall is enriched in type I and III fibrillar collagens, which are highly thrombogenic matrices, but this property may be influenced by other extracellular matrix components, for instance by proteoglycans. The proteoglycan distribution is different in distinct vessel layers, including mainly versican and decorin in the adventitia and versican, biglycan and perlecan in the media [9–15]. The media layer of human arterial vessel wall is resistant to platelet adhesion under high shear rate and the platelets adhere mainly to the adventitia [16]. However, after treatment with serine-proteases (trypsin, chymotrypsin and proteinase 3) or with chondroitinase ABC a seven- to tenfold increase in the VWF-mediated platelet adhesion to the collagen of the media is observed [16,17].

Adherent platelets recruit leukocytes from circulating blood, predominantly neutrophils, representing 76% of the leukocytes in thrombi [18] followed by activation of the thrombus-bound cells [19]. The initial interaction is P-selectin-dependent which binds to its leukocyte ligand PSGL-1 [20]. The interaction is strengthened by activation of an integrin receptor (α_M_ß_2_) via tyrosine phosphorylation mediated by src kinases that increases α_M_ß_2_-mediated binding to platelets [21]. Platelets also release microparticles with platelet-specific receptors and P-selectin, which promote platelet–platelet and platelet–leukocyte aggregation [22–24]. Thrombogenesis is supported by the platelet activating effects of cathepsin G released in the course of degranulation of the activated neutrophils together with neutrophil elastase [25,26]. Upon activation, neutrophils may release their secondary granule content including members of the metalloproteinase (MMP) family (interstitial collagenase, MMP-8 and gelatinase B, MMP-9) [27–30], but little is known about the potential impact of these proteases on the growing thrombus. The present study attempts to characterize the effects of neutrophils on the initial, VWF-dependent platelet adhesion to the vessel wall at a high shear rate.

## Methods

### Preparation of human artery cryosections

The study protocol was approved by the institutional and regional ethical board. Human iliac artery was removed from deceased healthy organ donors, immediately frozen in 2-methylbutane in dry ice and stored at −70 °C. Cryosections (6 μm thickness) of the artery were placed on poly-L-lysine-coated slides (‘Poly-Prep Slides’; Sigma-Aldrich Kft., Budapest, Hungary) 1–3 days before the experiments and stored at −20 °C until use.

### Model of platelet adhesion at high shear rate

The flow-chamber model described previously was used [31]. Artery cross-sections on poly-L-Lys-coated slides were perfused in a parallel-plate chamber with citrate-anticoagulated blood collected from healthy volunteers. A small parallel-plate chamber was constructed on the slides using a 0.3-cm-wide and 1.5-cm-long flow channel cut into a piece of double-sided tape (Scotch, 1.27 cm wide, 64 μm thick), which was sandwiched to a methacrylate cover (2.3 cm × 3.8 cm) containing the inlet and outlet tubing (1-mm internal diameter Tygon). The tissue section was positioned in the center of the flow channel between the inlet and outlet ports. Assuming laminar flow conditions, the shear rate at the surface of the section was 3350 s^−1^, according to the formula 1.03*6 *Q*/(w*h^*2*^), where *Q* is the flow rate in mL s^−1^, *w* and *h* are the width and the height of the flow path in cm, respectively [32]. This shear rate was chosen as an adequate model of the rheological situation in stenotic arteries [33]. Before the perfusion the sections were blocked with 2 w/v% bovine serum albumin (BSA) in 0.05 mol L^−1^ Tris buffer pH 7.4 containing 0.1 mol L^−1^ NaCl and 0.02 w/v% NaN_3_ (BSA-TBS) for 45 min. The 90-s perfusion was followed by a 30-s wash with 1.5 mmol L^−1^ KH_2_PO_4_, 8.1 mmol L^−1^ Na_2_HPO_4_ buffer pH 7.4 containing 137 mmol L^−1^ NaCl and 2.7 mmol L^−1^ KCl (PBS) with 13 mmol L^−1^ Na-citrate added. Thereafter, the sections were fixed in acetone at 4 °C for 10 min.

### Pretreatment of artery cryosections

Before perfusion cryosections were incubated with proteases, isolated polymorphonuclear (PMN) cells or the supernatant of the cells after 10-min activation with 1 μmol L^−1^ formyl-methionyl-leucyl-phenylalanine (fMLP; Sigma-Aldrich Kft). The cell suspension or protease solution was applied in a 100-μL volume on the surface of the cryosections and incubated for 30 min followed by washing three times with TBS. Enzyme concentrations were 10 nmol L^−1^ thrombin, 20 nmol L^−1^ neutrophil elastase, 17 nmol L^−1^ MMP-8 and 10 nmol L^−1^ MMP-9. Cell count was adjusted to 6000 μL^−1^ PMN cells in PBS containing 5 mmol L^−1^ glucose (PBS-glucose), which corresponds to the normal count in human blood [34]. MMPs secreted by PMN cells were activated with 1 mmol L^−1^ 4-aminophenylmercuric acetate (APMA) for 30 min at 37 °C.

### Visualization of adherent platelets

Platelets adherent to the tissue were visualized by indirect immunofluorescence microscopy. The sections were blocked with 200 μL BSA-TBS for 30 min followed by washing with TBS. Afterwards they were incubated for 60 min with 100 μL 4 μg mL^−1^ mouse monoclonal antibody against human CD41 GpIIb/IIIa (Biodesign International, Saco, ME, USA) diluted in BSA-TBS. The slides were washed three times with 50 mmol L^−1^ Tris-HCl, 100 mmol L^−1^ NaCl, 0.02 w/v% NaN_3_ pH 7.4 and 100 μL 2 μg mL^−1^ Alexa Fluor 488 goat-anti-mouse IgG (Invitrogen, Budapest, Hungary) in BSA-TBS was added and then incubated for 30 min. The slides were washed three times and glass coverslips were affixed over a drop of 50 v/v% glycerol in TBS. Confocal images were taken from the slides using a Zeiss LSM510 confocal laser scanning microscope equipped with a 20 × 1.4 objective (Carl Zeiss, Jena, Germany). The green fluorescent signal was acquired using a 488-nm excitation laser line (10% intensity) and emission was detected in the 500–530 nm wavelength range.

### Data analysis of platelet adhesion to the vessel wall

The quantification of platelets adhered to the vessel wall was performed with the Scion Image software (Scion Corp, Frederick, MD, USA) selecting the region of interest, calculating its surface area in pixels and setting a threshold intensity value for automatic identification of the area covered by adhered platelets. The area covered by platelets is reported in percentage of the whole area as mean ± SEM from *n =*5–32 images. To estimate the statistical significance of differences, Student’s two-sample *t*-test was used.

### Preparation of neutrophil granulocytes

Neutrophil granulocytes were isolated from the buffy coat fraction of human blood (Hungarian Blood Supply Service, Budapest, Hungary) [35], which was mixed with an equal volume of 2 w/v% Dextran T500 (GE Healthcare Bio-Sciences, Uppsala, Sweden) in saline followed by a centrifugation at 150 × *g* for 5 min. Platelet-rich supernatant was discarded and the residual fraction was mixed again with an equal volume of 2 w/v% Dextran T500 in saline and erythrocytes were allowed to sediment for 45 min. The supernatant was mixed with an equal volume of PBS-glucose and centrifuged for 3 min at 400 × *g*. The cell pellet was washed with an equal volume of PBS-glucose followed by centrifugation for 3 min at 400 × *g*. The PMN leukocyte-rich fraction was layered on an equal volume of Percoll (GE Healthcare Bio-Sciences) and centrifuged for 5 min at 400 × *g*. The supernatant was removed and further centrifuged for 15 min at 800 × *g*. The PMN-rich pellet was washed in PBS-glucose twice and centrifuged for 3 min at 400 × *g*. Isolated neutrophils were resuspended in PBS-glucose containing 1 mmol L^−1^ CaCl_2_. The cells were counted using an Abacus Junior B Hematology Analyser (Diatron, Budapest, Hungary). For activation of neutrophil granulocytes 1 μmol L^−1^ fMLP was used. MMP-release from isolated neutrophil granulocytes was measured with a Human Biotrak ELISA system for MMP-9 (GE Healthcare Bio-Sciences) according to the manufacturer’s instructions.

### Atomic force microscopy and scanning electron microscopy

Atomic force microscopy (AFM) images of native, protease- or neutrophil-treated non-fixed cryosections of arteries were acquired with a Nanoscope IIIa scanning probe microscope (Digital Instruments, Santa Barbara, CA, USA). The measurements were done in contact mode in air using cantilevers with a force constant of 0.12 N m^−1^ (Veeco Instruments Inc., Camarillo, CA, USA). Preparation, pre-treatment with various agents and washing of cryosections were performed as described above for the platelet adhesion procedure followed by storage at −20 °C until use. For scanning electron microscopy (SEM) evaluation the tissue samples were washed in 100 mmol L^−1^ Na-cacodylate buffer pH 7.2, followed by fixation for 10 min with 1 v/v% glutaraldehyde in the same buffer and dehydration in a dilution series of acetone and hexamethyldisilazane [36]. Before dehydration for enhancement of the proteoglycan structures in the SEM images the vessel wall cryosections were stained overnight with 0.05 w/v% Cupromeronic Blue (Sigma-Aldrich Kft) in 0.025 mol L^−1^ Na-acetate buffer pH 5.8 containing 2.5 v/v% glutaraldehyde and 0.3 mol L^−1^ MgCl_2_ [37]. The dried specimens were mounted on adhesive carbon discs, sputter coated with gold in SC7620 Sputter Coater (Quorum Technologies, Newhaven, UK) and images were taken with scanning electron microscope EVO40 (Carl Zeiss Gmbh, Jena, Germany). Sampling bias was excluded by repeating the measurements at least 10 times covering the whole surface of each cryosection for the AFM imaging and six times for the SEM imaging.

### Purified enzymes

Active enzyme concentration of human neutrophil elastase (Serva Electrophoresis Gmbh, Heidelberg, Germany) was determined as described previously [38]. Recombinant human MMP-8 and MMP-9 proenzymes (R&D Systems, Abingdon, England) were activated with 1 mmol L^−1^ p-aminophenylmercuric acetate (APMA; Sigma-Aldrich Kft) at 37 °C for 1 h and 24 h, respectively, and their activity was determined with gelatin substrate zymography [39]. Chondroitinase ABC (Sigma-Aldrich Kft) was used according to the preparation activity stated by the manufacturer. Bovine thrombin was purchased from Serva Electrophoresis Gmbh (Heidelberg, Germany), thrombin was further purified by ion-exchange chromatography on sulfopropyl-Sephadex yielding preparation with specific activity of 2100 IU mg^−1^ [40] and recombinant hirudin (Sigma-Aldrich Kft) was used for its inactivation. MMP2/MMP-9 Inhibitor I [(2R)-2-[(4-biphenylylsulfonyl)amino]-3-phenylpropionic acid] for the inhibition of MMP-9 and MMP-Inhibitor I (4-Abz-Gly-Pro-D-Leu-D-Ala-NHOH) for the inhibition of MMP-8 (Calbiochem, LaJolla, CA, USA) were applied in combination at 5 μmol L^−1^ concentration each. Pefabloc (aminoethyl-benzenesulfonylfluoride; Serva Electrophoresis Gmbh) was used for the inhibition of serine proteases at 100 μmol L^−1^ concentration.

## Results

**Fig. 1:**
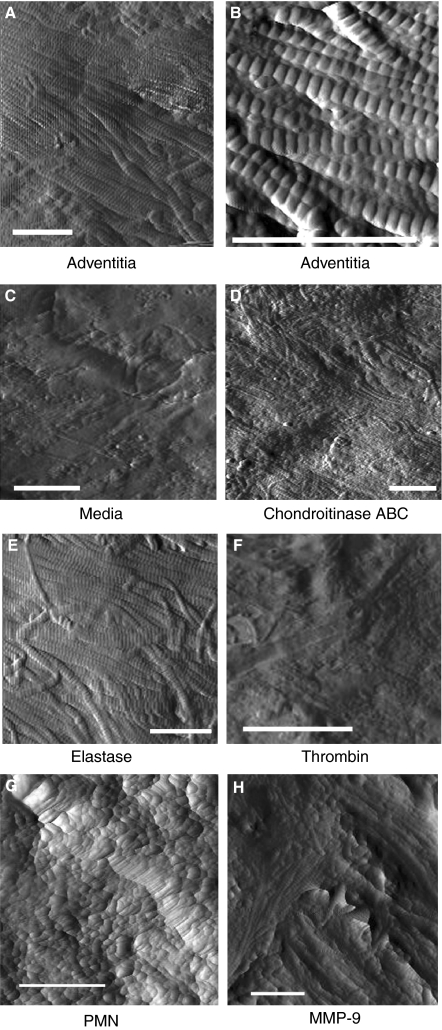
Human iliac artery imaged using contact mode atomic force microscopy (AFM). Artery cryosections were prepared and examined with AFM as described in Methods. A–C, native unfixed adventitia and media layers; D–H, media of cross-sections treated for 30 min with 4 nmol L^−1^ chondroitinase ABC (D); 20 nmol L^−1^ neutrophil elastase (E); 10 nmol L^−1^ thrombin (F); 6000 μL^−1^ polymorphonuclear (PMN) cells activated with 1 μmol L^−1^ formyl-methionyl-leucyl-phenylalanine (fMLP) and 1 mmol L^−1^ 4-aminophenylmercuric acetate (APMA) for 10 min prior the application to the artery cross-section (G) or 10 nmol L^−1^ MMP-9 (H). Scale bar = 1 μm. Each image is a representative of at least five sections.

**Fig. 2:**
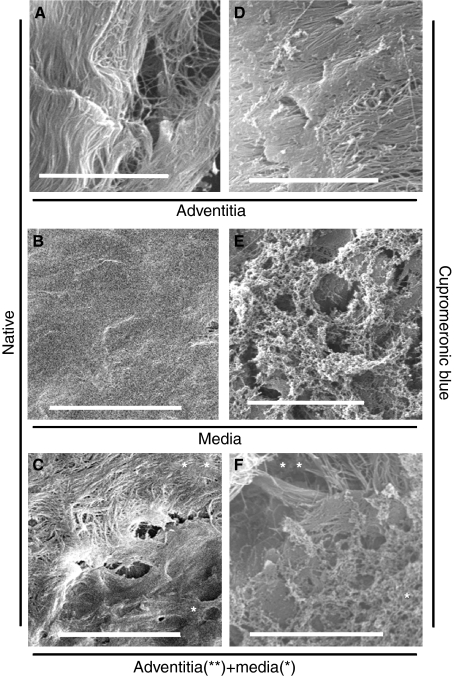
Human iliac artery imaged scanning electron microscopy (SEM). Artery cryosections were prepared for SEM examination as described in Methods. Native, untreated cross-sections are shown in panels A–C, whereas Cupromeronic Blue staining was used for enhancement of the proteoglycan visualization in panels D–F. Each image is a representative of at least five sections, in each of which six randomly selected fields were observed at three levels of magnification (1000×, 5000× and 10 000×).

**Fig. 3:**
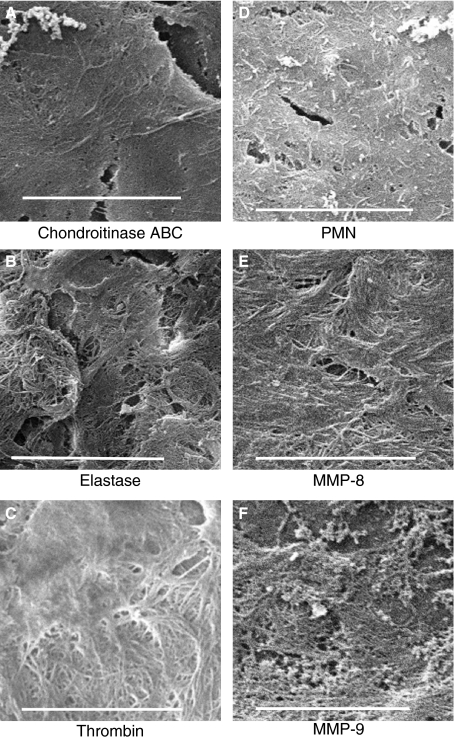
The media layer of pretreated human iliac artery imaged by scanning electron microscopy (SEM). Prior to staining with Cupromeronic Blue artery sections were treated for 30 min with 4 nmol L^−1^ chondroitinase ABC (A), 20 nmol L^−1^ neutrophil elastase (B), 10 nmol L^−1^ thrombin (C), 6000 μL^−1^ polymorphonuclear (PMN) cells activated with 1 μmol L^−1^ formyl-methionyl-leucyl-phenylalanine (fMLP) and 1 mmol L^−1^ 4-aminophenylmercuric acetate (APMA) as in Fig. 1 (D), 17 nmol L^−1^ MMP-8 (E), 10 nmol L^−1^ MMP-9 (F). Scale bar = 10 μm. Each image is a representative of at least five sections, in each of which six randomly selected fields of the media layer were observed at three levels of magnification (1000×, 5000× and 10 000×).

The changes in the vessel wall structure after exposure to leukocytes or leukocyte-derived proteases were monitored by high-resolution microscopy (AFM and SEM). AFM has the potential to achieve submolecular resolution under physiological conditions. Using this method, a finely pointed tip is moved over the surface by a piezoelectric scanner; the undulations of the tip are monitored with a laser-diode detector and translated into a three-dimensional topographical image. This technique was first applied to study the ultrastructure of collagen fibrils and their association with proteoglycans in human cornea and sclera [41,42]. The AFM images of native cryosections show a compact structure of the media, in which the collagen fibers are covered by a homogeneous amorphous layer, while in the adventitia the striped morphology of the collagen fibers is clearly visualized (Fig. 1A–C). The striped collagen structure in the media layer is seen only after enzymatic or cellular pre-treatment (Fig. 1D–H). In order to increase the electron density of proteoglycans and prevent the complete collapse of their structure, in SEM imaging we applied Cupromeronic Blue, a cationic dye of well-characterized specificity for proteoglycans and sulfated glycosaminoglycans [43], which has been widely used in transmission and scanning electron microscopy [44,45]. In native SEM images (Fig. 2A–C) the rough collagen bundle-arrangement in the adventitia stands in contrast with the bundle-free smooth surface of the media. Pre-treatment with non-activated PMN cells does not alter the media structure (not shown). Using Cupromeronic Blue staining proteoglycans can be directly visualized on the surface of collagen fibers in the media, but not in the adventitia (Fig. 2D–F). The Cupromeronic Blue staining is weak in the adventitia indicating that collagen bundles are hardly covered by proteoglycans, whereas the layer of the media shows a strong Cupromeronic Blue signal because of proteoglycan bunches. The specificity of Cupromeronic Blue staining for proteoglycans in the applied artery specimens was confirmed with chondroitinase ABC treatment, which is known to degrade proteoglycans and accordingly removes the proteoglycan-related SEM morphological signs, the fine granular staining in the media (compare Figs 2E and 3A). Activated neutrophil granulocytes, neutrophil-derived proteases (elastase, MMP-8, MMP-9) as well as thrombin also strip the proteoglycan meshwork off the media layer (Fig. 3B–F).

**Fig. 4:**
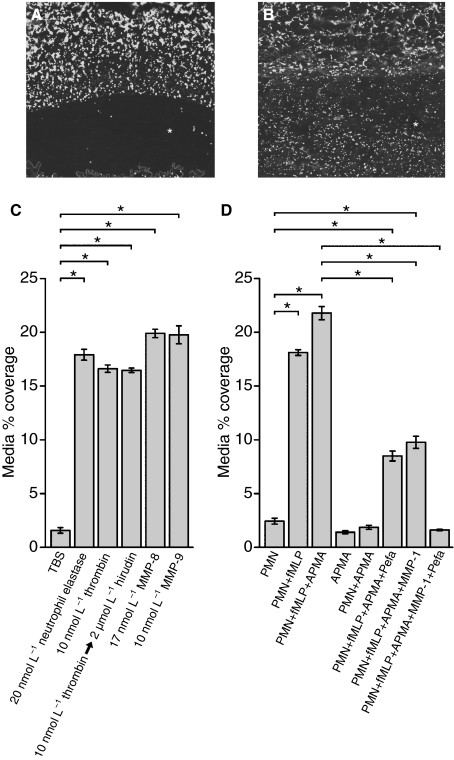
Platelet adhesion to distinct layers of the arterial wall. Human iliac artery sections were incubated for 30 min with Tris-buffered saline (A) or with PMN+fMLP+APMA (B, as an illustration of the treatments listed in panel C and D). Thereafter, artery specimens were perfused with citrated whole blood for 1.5 min at 3350 s^−1^ shear rate in the flow chamber described in Methods. Platelets were visualized by indirect immunofluorescence and images were analyzed for platelet coverage of the separate layers (the media layer is indicated by asterisk). In panels C and D the area in the media layer covered by platelets is shown in percentage of the total media area (mean ± SEM, *n*=5–32) for the same experimental setup, in which the 30-min treatment preceding the blood perfusion was performed with various agents as indicated (in the sample labeled ‘thrombin→hirudin’ the artery section was incubated for 30 min with hirudin after a 30-min treatment with thrombin). PMN, 6000 μL^−1^ PMN cells in PBS-glucose; fMLP, 10-min activation of the cells with 1 μmol L^−1^ fMLP prior the application to the artery cross-section; APMA, 1 mmol L^−1^ APMA; Pefa, 100 μmol L^−1^ Pefabloc; MMP-I, a mixture of 5 μmol L^−1^ MMP2/MMP-9 Inhibitor I and 5 μmol L^−1^ MMP-Inhibitor I. Asterisk indicates difference significant at *P* < 0.001 level.

When citrated whole blood is perfused over cross-sections of human iliac artery at a surface shear rate of 3350 s^−1^, platelets adhere mainly to the adventitia layer of the vessel wall and not to the media (Fig. 4A). Platelet adhesion in this model can be inhibited by an anti-VWF antibody, which blocks the VWF binding to collagen [17]. In native sections the platelet coverage of the adventitia is 23.39 ± 1.39%, while only 1.57 ± 0.25% of the media is covered by platelets. Pre-treatment of vessels with serine proteases at physiologically relevant concentrations (about 10 nmol L^−1^ thrombin is present in whole blood at the point of clotting [46], 20 nmol L^−1^ neutrophil elastase concentration is expected in cell suspension of degranulated PMNs [47–49]) promotes platelet adhesion to the media layer (Fig. 4C). The plasma protease inhibitors present at a micromolar concentration in the perfused whole blood preclude direct platelet effects of traces of protease from the pre-treatment and in line with this the thrombogenicity of adventitia is not affected by these treatments (not shown). If after treatment with 10 nmol L^−1^ thrombin the arterial section is exposed to 2 μmol L^−1^ hirudin for 30 min, the media coverage with platelets from whole blood circulated over the section is the same as in a thrombin-treated section without thrombin blockade (Fig. 4C). This control measurement excludes direct platelet recruitment by traces of thrombin in the pre-treated arterial sections, which could be hypothesized in light of the known platelet-activating effects of thrombin.

Pre-treatment with isolated and fMLP-activated neutrophil granulocytes increases the media platelet adhesion up to 18.11 ± 0.27% area coverage (Fig. 4D) and the supernatant of these cells yields similar coverage of the media (not shown). If MMP activator (APMA) is also present, the thrombogenic effect of activated neutrophils is enhanced (21.77 ± 0.61% platelet coverage in the media layer), whereas MMP-inhibitors (against MMP-8 and -9) significantly moderate it (9.77 ± 0.56% coverage). In the presence of a broad-spectrum serine protease inhibitor (Pefabloc) a similar moderate-level coverage induced by activated neutrophils is observed (8.49 ± 0.47%). The combined administration of MMP and serine protease inhibitors completely abolishes the effect of neutrophils on the platelet adhesion to the media (1.61 ± 0.07% coverage). Cells which were not activated with fMLP did not influence significantly the platelet adhesion to the media layer (2.43 ± 0.27% coverage). The release of MMP-9 from activated neutrophils was measured as an indicator of degranulation. After the same activation with fMLP as applied in the experiments shown in Fig. 4, a single neutrophil granulocyte secretes 0.075 pg MMP-9. Accordingly, the effects of purified neutrophil proteases (MMP-8, MMP-9, neutrophil elastase) were evaluated at concentrations relevant to such a degree of degranulation [47–49]. The enhancement of platelet adhesion to the media is reproduced by 17 nmol L^−1^ MMP-8, 10 nmol L^−1^ MMP-9 or 20 nmol L^−1^ neutrophil elastase (Fig. 4C).

## Discussion

A flow-chamber model, in which anticoagulated whole blood is perfused through a channel over cross-sections of arteries at a well-defined surface shear rate, was used as a tool to analyze the thrombin-independent phase of platelet deposition separately from the subsequent thrombin-dependent platelet adhesion. In this chamber the contact of circulating platelets with all arterial wall layers models the situation at sites of incision injury or atherosclerotic lesions [50] in blood vessels and allows exploration of the thrombogenic potential of distinct vessel wall layers. The application of arterial cryosections in this model approximates the native structure of the vessel wall avoiding gross mechanical perturbation of the wall architecture caused by stripping off the intima in other models [16]. In addition, the VWF-dependent nature of the platelet-vessel wall interactions under the experimental conditions of the present study is well characterized; an anti-VWF antibody, which blocks the VWF-collagen interaction, inhibits the platelet adhesion to native adventitia, as well as to chondroitinase ABC or acid-treated media [17]. The two-stage treatment mode applied in our experimental setup (pre-treatment of the vessel wall with PMN cells or enzymes followed by perfusion with whole blood) prevents promotion of platelet adhesion by direct interactions between platelets and PMN cells. In addition, the abundant protease inhibitors from the whole blood used in the second stage eliminate any traces of proteases remaining in the artery section from the first stage of the assay. Thus, the observed changes in platelet adhesion can be reliably attributed to the vessel wall modifications induced in the first stage of treatment in our model.

Our present data confirm that the thrombogenicity (platelet binding ability) of native arterial media at high shear rate is very poor (Fig. 4A) in agreement with earlier reports [17,50]. At sites of incision injury or ruptured atherosclerotic lesions this property may attenuate thrombus formation and thus a counteracting mechanism should operate for efficient platelet plug formation. Because PMN cells are major components of thrombi influencing both thrombus formation and lysis [51], we hypothesized a novel function of these cells in the initial stage of thrombus formation: structural perturbation of the thromboresistant media increasing its platelet-adhesive ability. Our data (Fig. 4C,D) indicate that a short (30-min) exposure of the media to activated neutrophil cells or their supernatants profoundly increases the adhesion of platelets to the media layer to a degree similar to that in the adventitia. Perfusion of the cryosection with these cells before perfusing with whole blood gave similar results concerning platelet adhesion. This increase in thrombogenicity is completely reversed by a combination of serine and metalloproteinase inhibitors (Fig. 4D) in agreement with a proteolytic mechanism underlying this cellular effect. Activated PMN cells secrete proteases, which belong to two main enzyme families, serine proteases (neutrophil elastase) and matrix metalloproteinases (MMP-8 and MMP-9). Most of these proteases remain membrane bound [52,53] and the tightly adherent cells create a microenvironment from which inhibitors are relatively excluded generating high local enzyme concentrations which overwhelm local defense mechanisms. The elastase concentration is millimolar in azurophil granules and exceeds the extracellular inhibitor concentrations by more than two orders of magnitude, thus its release creates a pericellular zone of active proteolysis [48,49]. The contribution of MMPs to thrombin-induced platelet-leukocyte aggregation is well characterized under static conditions [54], but little is known of their role at the interface of circulating blood and the vessel wall at a stage of thrombus formation that precedes blood coagulation. Our present findings show that partial proteolysis of the artery cross-sections with each of these leukocyte-derived proteases separately abolishes the thromboresistance of the media (Fig. 4C). The impact of protease inhibitors on PMN-induced platelet adhesion and the reproduction of the cell effects by isolated PMN-derived proteases confirm the proteolytic background of the thrombogenic changes in the media induced by PMN cells.

According to an earlier report [17] the platelet adhesion in our experimental setup is mediated by VWF binding to collagen. Because the role of the composition of the reactive matrix in platelet adhesion is well established [55], we sought the differences in platelet adhesion to the native media and adventitia layers, as well as to the protease-modified media in variations of structure and accessibility of the collagen fibers in the vessel wall. We approached the morphological features of the adhesive substrate in the arterial wall with SEM and AFM analysis of the artery cryosections. The basal state of the extracellular matrix in our observations (Figs 1 and 2) corresponds to the human aortic wall structures evidenced by SEM and AFM on cryofractured samples [56]. Both microscopic techniques confirm the free accessibility of collagen fibers in the adventitia (Figs 1A,B and 2A,C) in line with the high platelet-adhesive potential of this layer (Fig. 4A). In contrast, no definite fibrous pattern can be distinguished in the AFM images of native media (Fig. 1C) despite the known collagen content of this layer. The AFM technique could not resolve any further details in the architecture of the native media probably because of the adhesion between the AFM tip and the amorphous substance filling the inter-fiber space. After Cupromeronic Blue staining this amorphous layer of the native media is visualized as a fine network superimposed over the collagen fibers (Fig. 2E,F). Based on the proteoglycan specificity of the dye [37] and the effect of the chondroitinase ABC treatment (Fig. 3A), we conclude that this fine meshwork represents proteoglycans intertwining the collagen fibers. The applied cellular (activated PMN) and enzymatic treatments (elastase, MMP-8 and MMP-9), which remove this proteoglycan network from the media observed with SEM (Fig. 3B–F), expose the striped morphology of collagen fibers seen on the AFM images (Fig. 1E–H). These findings provide explanation for the thromboresistance of the native media and its proteolytic abolishment. Proteoglycans are likely to be removed from critical sites of collagen fibers responsible for VWF binding. Because of the complexity of the three-dimensional structure of the collagen fibers, the current information on the location of the VWF binding sites in collagen is based primarily on data gained with synthetic triple-helical peptides, which map a single binding site to the D2 period of human collagen III [57]. Computational and molecular visualization models of the native collagen architecture clearly show that the proteolytic cleavage [58] or ligand-interaction sites [59] in collagen may express variable accessibility for proteases or ligands. Thus, in the context of the arterial wall, proteoglycans in the media could easily mask the VWF binding sites on the surface of collagen preventing platelet adhesion at high shear rates, whereas partial proteolysis exposes these sites for interaction with VWF. Promotion of VWF-collagen interaction does not require complete removal of the proteoglycan matrix as evidenced by the thrombin effects. Although thrombin causes only focal exposure of the collagen fibrils in the media (Fig. 1F), platelet adhesion is significantly increased (Fig. 4C). The modification of the vascular structure represents a novel aspect of the thrombin role in platelet plug formation. While thrombin-mediated activation of platelets is well characterized [60], no data are available on its impact on the extracellular matrix of the vessel wall.

In summary, our present data support the concept that neutrophil-related proteolysis reverses the thromboresistance of the native media layer of arteries and renders deeper vessel wall layers platelet adhesive at sites exposed to blood (e.g. in ruptured atherosclerotic plaques or incision injuries). Appreciation of the *in vivo* role of this mechanism requires future studies, which can substantiate new strategies for prevention of atherothrombosis.
